# Tobacco and cannabis use in the young ‘Not in Employment, Education or Training’ population: A systematic review and meta‐analysis

**DOI:** 10.1111/add.70477

**Published:** 2026-06-01

**Authors:** Clara Eyraud, Claire Collin, Philippe Martin, Corinne Alberti, Laëtitia Minary, Agnès Dumas, Enora Le Roux

**Affiliations:** ^1^ Université Paris Cité, INSERM ECEVE Paris France; ^2^ AP‐HP, Nord – Université Paris Cité, INSERM, Hôpital Universitaire Robert Debré Clinical Epidemiology Unit, CIC 1426 Paris France; ^3^ Institut National d'Etudes Démographiques (INED), UR14 – Sexual and Reproductive Health and Rights Aubervilliers France; ^4^ Université de Lorraine INSPIIRE Nancy France; ^5^ Inserm, Aix Marseille Université, Institut de Recherche pour le Développement (IRD), Institut des sciences de la santé publique (ISSPAM), Sciences Economiques et Sociales de la Santé et Traitement de l'information Médicale (SESSTIM), CALIPSO Team Marseille France; ^6^ University Center for Adolescent and Young Adult Health Fondation Santé des Etudiants de France Paris France

**Keywords:** cannabis use, meta‐analysis, NEET, systematic review, tobacco use, youth

## Abstract

**Background and aims:**

NEET (Not in Employment, Education or Training) youth represent a vulnerable population from a public health perspective, facing multiple health challenges, including elevated substance use. Tobacco and cannabis are the most commonly used psychoactive substances among young people, with early initiation associated with long‐term health and social consequences. While evidence suggests associations between NEET status and substance use, data remain limited regarding patterns of use and potential variations across different NEET profiles. This study aimed to characterise tobacco and cannabis use within the NEET population, taking into account the diversity of profiles, and to compare it with that of the general population, including employed youth and students, in order to inform targeted prevention strategies.

**Methods:**

A systematic review and meta‐analysis included observational studies examining tobacco and cannabis use among NEET youth aged 15–29 years. A search was conducted for observational studies available on PubMed, PsycINFO, Cairn and Web of Science databases and published between 1999 and 2025. Analyses studied the variations in use among NEET profiles and compared NEET against employed youth, student and general population controls. Random‐effects models generated pooled crude odds ratios. Sensitivity and subgroup analyses were conducted based on study quality, gender and type of comparison population.

**Results:**

Twenty‐five studies were included and analysed, including a total of 91 085 individuals. A statistically significant association between NEET status and both tobacco use [odds ratio (OR) = 1.92, 95% confidence interval (CI) = 1.46–2.53] and current cannabis use (OR = 2.14, 95% CI = 1.68–2.71) was found. Current smoking was statistically significantly more prevalent among NEET than among students (OR = 3.05, 95% CI = 2.31–4.03), but not statistically significantly different compared with young workers (OR = 1.10, 95% CI = 0.91–1.34). NEET demonstrated statistically significantly higher cannabis use compared with both students (OR = 1.81, 95% CI = 1.34–2.44) and workers (OR = 1.67, 95% CI = 1.21–2.31). A higher prevalence of cannabis use disorder among young people with NEET status (31.93%) was observed compared with their non‐NEET peers (12.12%), but no statistically significant association was found.

**Conclusions:**

Youth Not in Employment, Education or Training (NEET) appear to show higher tobacco and cannabis use compared with their peers. Given these findings, targeted prevention strategies addressing substance use in NEET populations are essential to reduce social health inequities.

## INTRODUCTION

Following the 2008 financial crisis, the transition from education to the labour market became increasingly challenging for youth [1]. In light of this context, a new term was adopted to better measure the complexity of youth unemployment. The term NEET (Not in Employment, Education or Training) was introduced to designate individuals aged 15–29 years who are neither working nor enrolled in any educational or training programme [2].

Young people with NEET status represented 11.2% of those aged 15–29 years in Organisation for Economic Co‐operation and Development (OECD) member countries in 2024 [3]. According to Eurofound, in 2016 in Europe, the NEET category was made up of seven distinct profiles with varying distributions: short‐term unemployed (29.8%); long‐term unemployed (22.0%); those with family responsibilities (15.4%); re‐entrants (hired or enrolled, soon exiting NEET status) (7.8%); individuals with illness or disability (6.8%); discouraged workers (who believe there are no job opportunities and have stopped looking for work) (5.8%); and ‘other NEET’ (heterogeneous subgroup including all individuals who do not fall into the above categories, e.g. street person, unpaid artistic careers) (12.5%) [4].

Although the NEET concept has been used as a policy indicator since the 1990s [2], research examining health behaviours within this population has only emerged in recent years. Originally developed as an economic indicator, it has been increasingly used in public policy, notably through European initiatives aimed at supporting young people not in employment, education or training (e.g. the launch of the Youth Guarantee in 2013). Concurrently, empirical research examining the social and health vulnerabilities associated with NEET status has grown substantially, particularly over the past decade. This expansion coincides with rising NEET rates following the 2008 economic crisis and renewed policy attention towards youth disengagement during the COVID‐19 pandemic [5, 6]. This meta‐analysis therefore consolidates this emerging evidence base.

Tobacco and cannabis are among the most frequently used psychoactive substances in youth populations [7, 8]. According to a 2021–2022 World Health Organization (WHO) report, 15% of 15‐year‐olds in Europe, Canada and Asia had already smoked tobacco, and 12% had used cannabis [8]. Tobacco and cannabis use are interconnected, with each substance increasing the likelihood of using the other [9, 10]. The initiation of tobacco and cannabis use at an early age is also a risk factor for becoming a regular smoker in adulthood [11, 12] and increases the risk of nicotine and cannabis dependence [13, 14]. Tobacco and cannabis use are also associated with significant long‐term health consequences [7, 11]. Tobacco use is linked to cardiovascular disease, neoplasms and chronic respiratory diseases [15], while cannabis use is associated with the development of cognitive and psychiatric disorders [16, 17, 18].

Evidence also highlights a bidirectional relationship between substance use and employment: substance use has been shown to hinder access to employment and is associated with prolonged periods of joblessness [19]. Reciprocally, unemployment is a significant risk factor for both initiating and maintaining substance use [20].

This bidirectional association is especially salient among young people with NEET status, a population facing heightened health vulnerability [21, 22, 23]. The literature has reported that they experience higher rates of substance use and poorer mental health than their peers [21, 24]. A meta‐analysis has established significant associations between NEET status and substance use disorders, notably cannabis [25, 26]. However, little is known about the potential differences in tobacco and cannabis use across NEET profiles (e.g. unemployed, with family responsibility or with disabilities) as categorised by Eurofound, and how the estimated risk of substance use varies depending on which comparison group is selected among same‐age youth. Understanding these variations is essential for developing targeted and effective prevention and care strategies.

The NEET population may comprise distinct subgroups associated with differential risks for substance use, and the choice of comparison group (students vs employed youth vs general population) may substantially influence risk estimates. The aim of this study was to investigate tobacco and cannabis use within the NEET population, incorporating these diverse profiles, and comparing this with the general population, including employed youth and students.

## METHODS

This systematic review was conducted following the Preferred Reporting Items for Systematic reviews and Meta‐Analyses (PRISMA) [27] and Meta‐analyses of Observational Studies in Epidemiology (MOOSE) [28] guidelines (Appendix S1). The study protocol has been registered in PROSPERO (CRD42023401994).

### Eligibility criteria

Eligible studies met the following inclusion criteria: (i) included a population aligned with the Eurofound definition of NEET status [4], a population explicitly defined as NEET (NEET‐identified) or a population of NEET‐related subgroups (e.g. unemployed, school dropouts), aged 15–29 years (i.e. age range or mean/median within this range); (ii) examined the relationship between substance use (i.e. tobacco, cannabis, alcohol or other drugs) and NEET status; (iii) were observational and included a comparison group (young people of the same age, whether employed or studying); (iv) were published between January 1999 and July 2025, as the term NEET was first used in 1999 [2], in French or English.

The review initially included all substances. Illicit drugs and medications were excluded because the limited number of articles and the wide variety of substances and definitions of their use did not allow for a relevant meta‐analysis. The analyses ultimately focused exclusively on alcohol, tobacco and cannabis. For this meta‐analysis, only data related to tobacco and cannabis were used, as both substances share the same mode of administration and similar ways of measuring consumption (e.g. occasional or daily smoking). Moreover, none of the included studies differentiated between pure cannabis and cannabis mixed with tobacco. Given that mixed use represents the predominant form of cannabis consumption [9, 10], treating tobacco and cannabis separately would not have been meaningful. In addition, tobacco and cannabis use have often been associated with the same coping motives, such as managing stress or negative emotions, whereas alcohol use among young people has more frequently been linked to social motivation [29, 30].

Substances that share similar modes of administration or measurement methods, such as e‐cigarettes or nitrous oxide, were searched for but no studies on these substances met the eligibility criteria for this review.

### Search strategy

The search strategy was implemented in PubMed (epidemiology and medicine), PsycINFO (psychology and addiction), Cairn (French‐language publications in humanities and social sciences) and Web of Sciences (multidisciplinary health and social sciences). The search strategy grouped terms related to the target population (e.g. ‘NEET’ or ‘unemployed’) and referring to consumption (e.g. ‘substance use’, ‘smoker’ or ‘cannabis’) (Appendix S2). We also screened the reference lists of the selected studies and previous systematic reviews and meta‐analyses to identify additional relevant studies.

### Study selection

Two researchers (C.E. and C.C.) independently screened titles and abstracts, then full texts for inclusion. Any disagreements about inclusion were resolved by a third researcher (E.L.R. or P.M.). Covidence systematic review software (Veritas Health Innovation, Melbourne, Australia) was used to select and include articles, as well as to extract data. When multiple publications used the same data set, we included only the study with the largest sample size and highest methodological quality to prevent data duplication.

### Data extraction

Data extraction was performed following the same procedure as the study selection process. Two researchers (C.E. and C.C.) independently extracted data, and the calculations required for the meta‐analysis were performed independently and progressively compared and reconciled as they were completed. In case of disagreement regarding extraction, the two researchers first sought to reach consensus through discussion. Only persistent cases were resolved by a third researcher (E.L.R. or P.M.).

Data were extracted using a standardised form that captured: (i) study methodology and main characteristics; (ii) population characteristics (NEET status, age, sex and other socio‐demographic characteristics, when available); and (iii) substance use definition, rate and statistical association in populations (measure definition, measurement instrument, prevalence, crude and adjusted odds ratio).

### Quality assessment

The quality of individual studies was assessed using Standard Quality Assessment Criteria (QualySyst) [31] for the cross‐sectional studies and with the Newcastle–Ottawa Scale (NOS) [32] for cohort studies. Studies were rated as ‘strong’ (QualySyst)/‘good’ (NOS), ‘medium’ (QualySyst)/‘fair’ (NOS) or ‘weak’ (QualySyst)/‘poor’ (NOS). Studies rated as ‘weak’ or ‘poor’ were excluded from the meta‐analysis (Appendix S3).

An assessment of the quality of evidence was conducted using the GRADE instrument [33] (Appendix S4).

### Statistical analysis

#### Tobacco and cannabis consumption categorisation

The analysis focused on three standardised categories of tobacco and cannabis use commonly reported in the literature.

- Current tobacco use: characterised using the definition of ‘current smoker’, in accordance with the Centers for Disease Control and Prevention, namely a person who currently smokes cigarettes (daily smoker or someday smoker) [34].
- Cannabis use was examined through two variables: (i) current cannabis use, defined as consumption in the past month (or past 3 months when monthly data are unavailable, with sensitivity analysis conducted to assess potential bias) according to major European and American population surveys [35, 36, 37]; and (ii) cannabis use disorder (CUD), defined as the presence of clinically significant impairment or distress within 12 months according to the Diagnostic and Statistical Manual of Mental Disorders, 5th Edition (DSM‐5) [38]. Studies that did not use DSM‐5 criteria for CUD were classified under current use.
- Other patterns of tobacco or cannabis consumption (experimentation, occasional use) were not analysed.

#### Population categorisation

The analyses considered two main categories of young people with NEET status.

- NEET‐identified: population explicitly labelled as ‘NEET’ by study authors, bringing together different NEET profiles, such as unemployed or out of labour force (primarily comprising homemakers and caregivers);
- NEET‐related subgroups: populations not explicitly labelled as ‘NEET’ by the study authors but meeting the Eurofound definition [4] (e.g. unemployed, school dropout or living with a disability).

Additional analyses were conducted combining both categories (labelled as ‘all NEET’).

Comparisons were made against three reference groups: general youth population; employed youth only; and students only.

#### Descriptive statistics

For descriptive statistics, results were expressed as numbers and percentages. Statistical analysis was conducted using Jamovi 2.5 (2024). Missing data were presented as not reported (NR).

#### Meta‐analyses

Three meta‐analyses were conducted to examine the associations between NEET status and tobacco use, current cannabis use and CUD. For each meta‐analysis, crude odds ratios (cORs) from individual studies were synthesised. For the NEET‐related subgroups, when multiple subgroups (e.g. unemployed, out of labour force) were present in a single study, they were pooled into a single cOR for the meta‐analysis. Missing cORs were calculated from available data, and corresponding authors were contacted when necessary to obtain unpublished data.

A random‐effects model was employed to compute pooled cORs and 95% confidence intervals. The weight of each study was calculated using the inverse‐variance method. Heterogeneity between studies was assessed using multiple indicators: τ^2^, *I* ^2^ statistic (with heterogeneity threshold set at 25% [39]) and Cochran’s *Q*‐test (significance level *P* < 0.05). Results were visually presented using forest plots. All statistical analyses were performed using Cochrane Review Manager (RevMan 8.8.0) (The Cochrane Collaboration, October 2024).

To evaluate the robustness of the findings, a sensitivity analysis was performed on studies that were strictly classified as ‘strong’ (QualySyst) or ‘good’ (NOS) quality. Additionally, when sufficient data were available, subgroup analyses were conducted to investigate potential sources of heterogeneity, stratifying results by gender and comparison populations (students, employed or general population). An analysis integrating current cannabis use and CUD was also performed to examine whether their combination yielded results consistent with those obtained when each was studied separately.

## RESULTS

**FIGURE 1:**
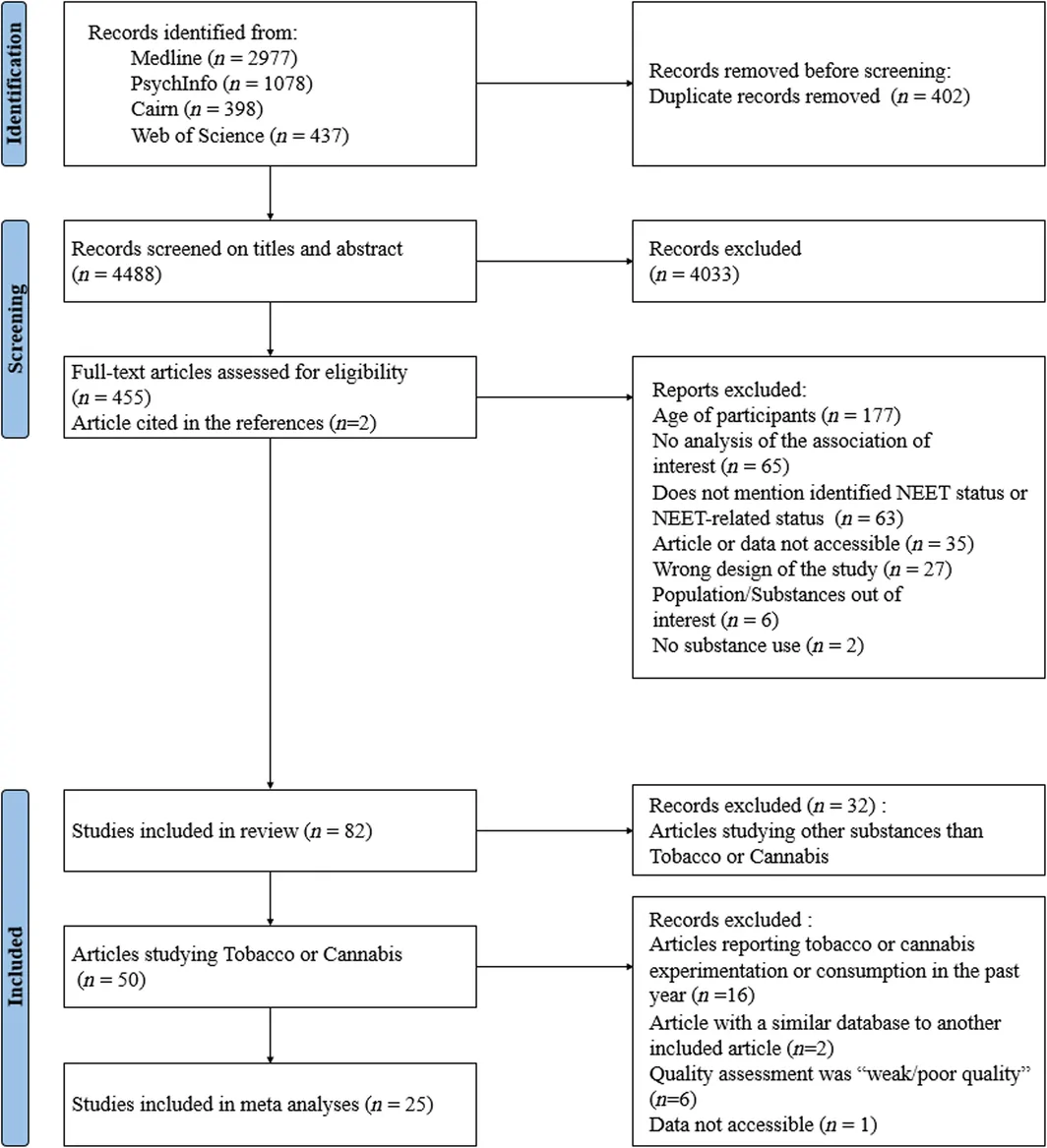
Flow chart of study identification and selection.

Fifty articles studying tobacco and cannabis use were identified. Of those, 25 were secondarily excluded for the following reasons: reporting on other patterns of tobacco or cannabis consumption (i.e. experimentation, occasional use) (*n* = 16); presenting results derived from overlapping databases (*n* = 2); or the data reported in the articles were not accessible (*n* = 1) or were rated as being of weak/poor methodological quality (*n* = 6). This resulted in 25 studies included in the meta‐analyses (Figure 1). Quality assessment demonstrated that 80% (20/25) of the included studies met the criteria for strong methodological quality.

Thirteen studies examined tobacco use only, six focused exclusively on current cannabis use and one focused on CUD. Three studies investigated both tobacco and current cannabis use within the same article, while two addressed tobacco and CUD. Thus, in total, the associations between NEET status and tobacco use, current cannabis use and CUD were examined in 18, 9 and 3 studies, respectively (Table 1).

**TABLE 1:** Characteristics of the 25 articles included in the meta‐analyses.

	*n* articles (%)			
	Current tobacco use	Current cannabis use	CUD	All articles
	18 (100)	9 (100)	3 (100)	25 (100)
**Geographical area**				
Europe	12 (67)	4 (45)	3 (100)	14 (56)
North America	3 (17)	3 (33)	0 (0)	6 (24)
Oceania	1 (6)	2 (22)	0 (0)	3 (12)
Asia	2 (11)	0 (0)	0 (0)	2 (8)
**Publication date**				
2004–2008	3 (17)	1 (11)	0 (0)	3 (12)
2009–2013	3 (17)	2 (22)	1 (33)	5 (20)
2014–2018	5 (28)	3 (33)	1 (33)	7 (28)
2019–2023	7 (39)	3 (33)	1 (33)	10 (40)
**Study design**				
Cross‐sectional	11 (61)	3 (33)	1 (33)	14 (56)
Cohort	7 (39)	6 (67)	2 (67)	11 (44)
**Population recruitment**				
National survey	12 (67)	4 (44)	3 (100)	15 (60)
Health structure	2 (11)	2 (22)	0 (0)	3 (12)
Employment/educational structure	4 (22)	3 (33)	0 (0)	7 (28)
**Measurement instruments**				
ASSIST	0 (0)	1 (11)	0 (0)	1 (4)
NSDUH tool	0 (0)	2 (22)	0 (0)	2 (8)
Young Minds Matter tool	1 (6)	0 (0)	0 (0)	1 (4)
CAST	0 (0)	0 (0)	1 (33)	1 (4)
DSM‐IV	1 (6)	0 (0)	1 (33)	1 (4)
Not reported	16 (89)	6 (67)	1 (33)	19 (76)
**Quality assessment**				
Strong/good	14 (78)	7 (78)	2 (67)	20 (80)
Moderated/fair	4 (22)	2 (22)	1 (33)	5 (20)

Abbreviations: ASSIST = Alcohol, Smoking and Substance Involvement Screening Test; CAST: Cannabis Abuse Screening Test; CUD = cannabis use disorder; DSM‐IV = Diagnostic and Statistical Manual of Mental Disorders, fourth edition NSDUH = National Survey on Drug Use and Health.

### Study population description

This study includes 20 266 young people with NEET status (all NEET) comprising 6716 young people with NEET‐identified status and 13 550 young people with NEET‐related subgroup status. The NEET‐related subgroups (*n* = 13 550) comprised unemployed youth (*n* = 13 249), school dropouts (*n* = 231) and individuals out of the labour force (excluding students) (*n* = 70). No other profiles were found for the NEET‐related subgroups (Table 2).

**TABLE 2:** Population included in the meta‐analyses and their gender distribution (*n* = 90 856).

	Population of interest, *n* (%)					Population of comparison, *n* (%)				
	NEET‐identified	NEET‐related			Total (all NEET)	Non‐NEET	Employed	Student	Student and worker	Total
	NEET	Unemployed	Dropout	OLF^a^						
**Current tobacco use**										
Articles, *n*	9 (50)	8 (44)	1 (6)	1 (6)	18	9 (50)	8 (44)	7 (39)	NA	18
Population, *n*	6380 (32.97)	12 769 (65.99)	130 (0.67)	70 (0.36)	19 349	25 425 (38.82)	33 190 (50.68)	6879 (10.50)	NA	65 494
Gender, *n* articles^b^	6 (46)	6 (46)	1 (8)	1 (8)	13	5 (38)	6 (46)	7 (54)	NA	13
Men	1121 (18.98)	4675 (79.16)	79 (1.34)	31 (0.52)	5906	8468 (59.54)	2563 (18.02)	3192 (22.44)	NA	14 223
Women	1125 (13.94)	6852 (84.94)	51 (0.63)	39 (0.48)	8067	22 399 (77.07)	2977 (10.24)	3687 (12.69)	NA	29 063
Current cannabis use										
Articles, *n*	5 (56)	4 (44)	1 (11)	1 (11)	9	4 (44)	5 (56)	4 (44)	1 (11)	9
Population, *n* individuals	628 (39.37)	796 (49.91)	101 (6.33)	70 (4.39)	1595	7357 (52.52)	3118 (22.26)	3252 (23.21)	280 (2.00)	14 007
Gender, *n* articles^b^	2 (33)	4 (44)	NA	1 (11)	6	2 (33)	4 (44)	2 (33)	NA	6
Men	369 (48.30)	364 (47.64)	NA	31 (4.06)	764	5328 (67.89)	1447 (18.44)	1073 (13.67)	NA	7848
Women	88 (15.15)	454 (78.14)	NA	39 (6.71)	581	911 (21.11)	1811 (41.97)	1593 (36.92)	NA	4315
Cannabis use disorder										
Articles, *n* articles	1 (33)	2 (67)	NA	NA	3	1 (33)	2 (67)	2 (67)	NA	3
Population, *n*	239 (32.87)	488 (67.13)	NA	NA	727	1827 (45.66)	1621 (40.51)	553 (13.82)	NA	4001
Gender, *n* articles^b^	1 (33)	2 (67)	NA	NA	3	1 (33)	2 (67)	2 (67)	NA	3
Men	108 (22.88)	364 (77.12)	NA	NA	472	873 (35.01)	876 (35.12)	745 (29.87)	NA	2494
Women	131 (59.01)	91 (49.99)	NA	NA	222	954 (63.30)	335 (22.23)	218 (14.47)	NA	1507
All articles										
Articles, *n*	12 (48)	11 (44)	2 (8)	1 (4)	25	11 (44)	12 (48)	10 (40)	1 (4)	25
Population, *n*	6716 (33.20)	13 249 (65.50)	231 (1.14)	70 (0.35)	20 266	26 489 (37.40)	34 513 (48.73)	8984 (12.69)	833 (1.18)	70 819
Gender, *n* articles^b^	7 (37)	9 (47)	1 (5)	1 (5)	19	6 (32)	9 (47)	8 (42)	NA	19
Men	1198 (23.43)	5080 (77.57)	79 (1.24)	31 (0.49)	6388	9330 (35.01)	3341 (35.12)	3900 (29.87)	NA	16 571
Women	1213 (49.25)	6916 (51.75)	51 (0.62)	39 (0.47)	8219	23 310 (63.30)	3046 (22.23)	3840 (14.47)	NA	30 196

^a^ OLF = out of labour force; articles were excluded if they included students within their ‘out of labour force’ definition, as this contradicts the core NEET criteria that exclude those in education.

^b^ Gender: number of articles providing a detailed breakdown of gender within the socio‐professional categories.

The comparison population (*n* = 70 819) consisted of non‐NEET individuals from the general population of the same age range (*n* = 26 489), employed youth (*n* = 34 513), students (*n* = 8984) and individuals who were both students and employed (*n* = 833).

The gender composition of substance users was relatively consistent across NEET and non‐NEET groups for each behavior. Women represented a higher proportion of current tobacco users (57.7% in NEET, 67.1% in non‐NEET), while men predominated in current cannabis use (56.8% and 64.5%, respectively) and CUD (68.0% and 62.3%, respectively).

Across all studies, only one included the full NEET age range [7, 15, 16, 17, 18, 19, 20, 21, 22, 23, 24, 25, 26, 27, 28]. The remaining studies consistently covered at least part of the 15–25‐year age group, with nine articles including participants older than 25 years and six studies including people strictly under 21 years (Appendixes S5 and S6).

### Current tobacco use meta‐analysis

The meta‐analysis of 18 studies on young people with NEET status revealed that current tobacco use prevalence rates varied across different subgroups: 43.37% (*n* = 1105/2548) among NEET‐identified and 30.40% (*n* = 3949/12 989) among NEET‐related subgroups, with specific rates of 30.20% (*n* = 3857/12 769) among unemployed youth, 27.14% (*n* = 19/70) among individuals out of the labour force (excluding students) and 56.15% (*n* = 73/130) among high school dropouts.

**FIGURE 2:**
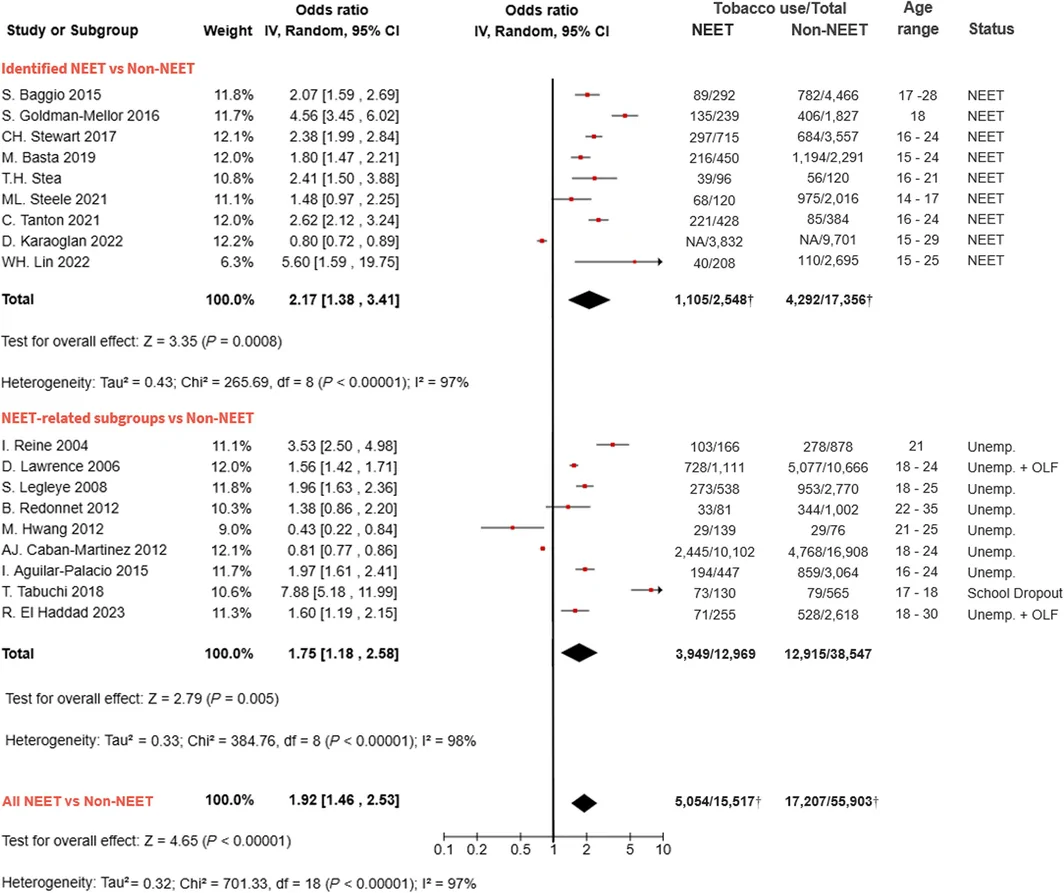
Meta‐analysis of the prevalence of current tobacco use among young people with NEET status: studies with more weight (large sample, low uncertainty) contribute more to the pooled OR; †calculated without article with missing data. Abbreviations: OLF = out of labour force; NA = missing data; NEET = not in employment, education or training; Unemp. = unemployed.

Current tobacco use was significantly associated with young people with NEET status in 14 studies. Pooled crude odds ratios indicate a higher risk of current tobacco use among young people with NEET status compared with their non‐NEET peers (OR = 1.92, 95% CI = 1.46–2.53). These associations were characterised by high statistical heterogeneity (*I* ^2^ = 97%) (Figure 2).

### Cannabis use meta‐analysis

Meta‐analysis of nine studies on young people with NEET status revealed that current cannabis use prevalence rates varied across different subgroups: 27.55% (*n* = 173/628) among NEET‐identified and 16.65% (*n* = 161/967) among NEET‐related subgroups, with specific rates of 21.78% (*n* = 22/101) among school dropouts, 16.76% (*n* = 129/796) among unemployed youth and 14.29% (*n* = 10/70) among individuals out of the labour force (excluding students). Descriptive analysis demonstrated higher prevalence rates among young people with NEET status (*n* = 334/1595, 20.94%) compared with their non‐NEET peers (*n* = 1598/14007, 11.41%).

**FIGURE 3:**
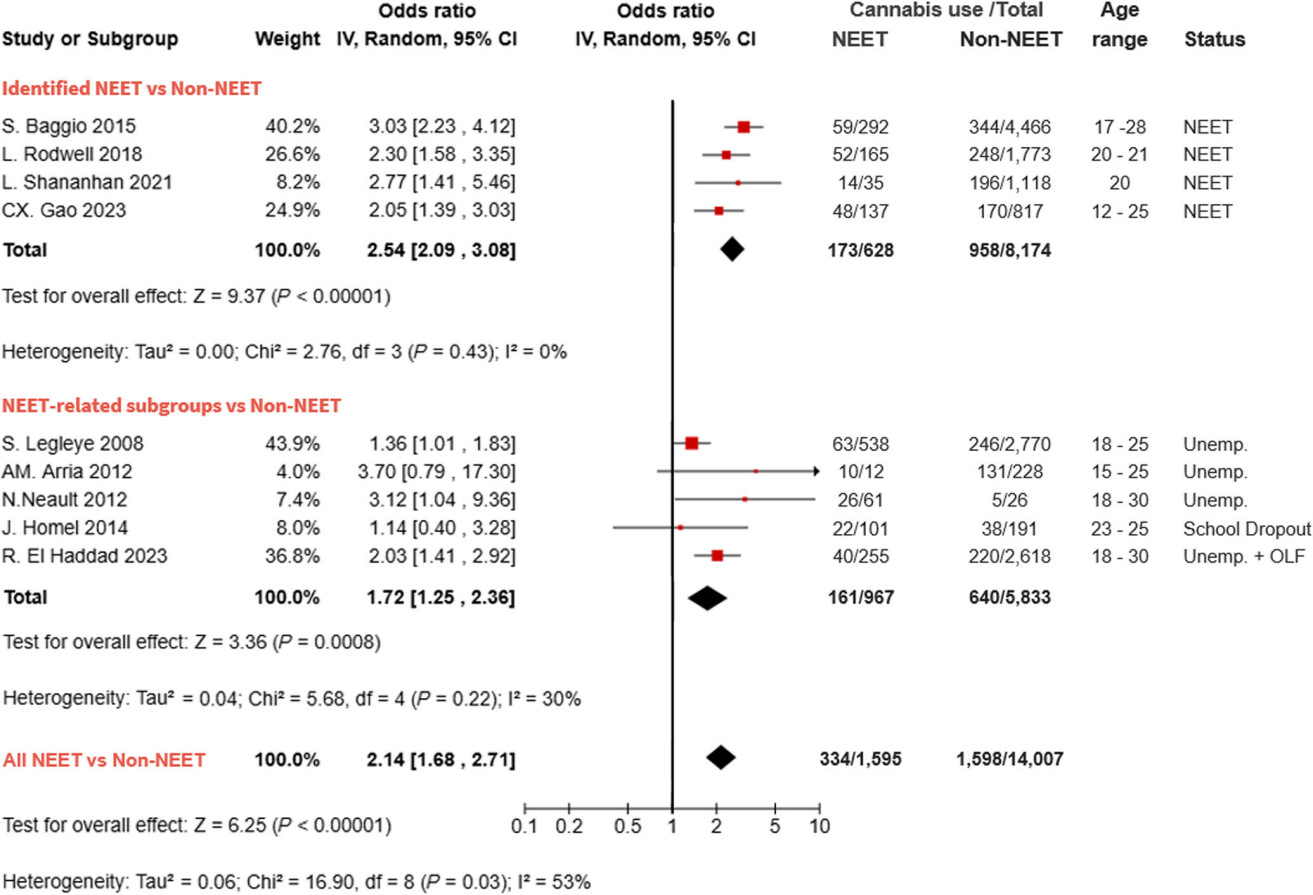
Meta‐analysis of the prevalence of current cannabis use among young people with NEET status: studies with more weight (large sample, low uncertainty) contribute more to the pooled OR. Abbreviations: OLF = out of labour force; NEET = not in employment, education or training; Unemp. = unemployed.

Significant associations between current cannabis use and NEET status were reported in all nine studies. The meta‐analysis revealed an overall increased risk of current cannabis use among young people with NEET status (OR = 2.14, 95% CI = 1.68–2.71). Statistical heterogeneity was moderate (*I* ^2^ = 53%) (Figure 3).

### Cannabis use disorder meta‐analysis

**FIGURE 4:**
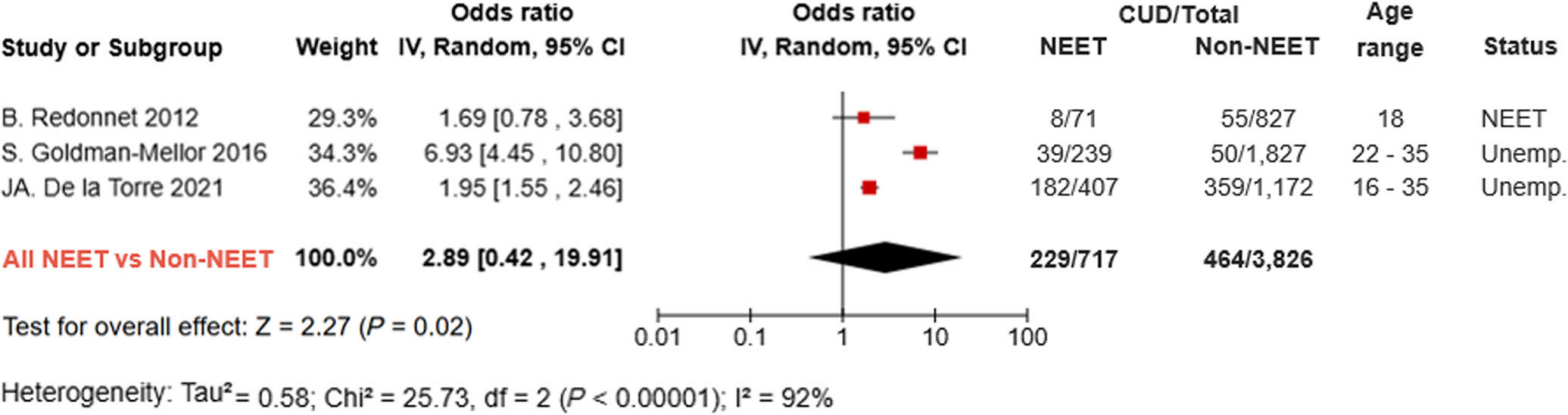
Meta‐analysis of the prevalence of cannabis use disorder among young people with NEET status: studies with more weight (large sample, low uncertainty) contribute more to the pooled OR. Abbreviations: CUD = cannabis use disorder; OLF: out of labour force; NEET: not in employment, education or training; Unemp.: unemployed.

Three studies examined associations between CUD and NEET status. The findings indicate a higher prevalence of CUD among young people with NEET status (*n* = 229/717, 31.93%) compared with their non‐NEET peers (*n* = 464/3826, 12.12%). However, the meta‐analysis did not reveal a related significant association (OR = 2.89, 95% CI = 0.42–19.91), with substantial heterogeneity observed (*I* ^2^ = 92%) (Figure 4).

### NEET profiles and their tobacco and cannabis use

Among the 12 studies reporting NEET‐identified data, four characterised profiles within their samples. Two of these applied Eurofound’s classification framework, identifying unemployed youth (48.98%), economically inactive individuals (32.20%), unpaid family business workers (10.92%), sick or disabled individuals (2.71%), those awaiting pre‐arranged employment (2.37%), and those pursuing other activities (2.80%).

Among these two studies using the Eurofound classification, only one analysed current tobacco use in relation to specific NEET profiles, focusing specifically on unemployed NEET individuals. This study found a significant association between current tobacco use and unemployed NEET status (OR = 2.34, 95% CI = 1.85–2.96) compared with non‐NEET individuals (Appendix S7). No studies examined cannabis use variations across NEET profiles compared with non‐NEET individuals.

### Sensitivity analysis

A sensitivity analysis including only high‐quality studies showed no change in the conclusions (Appendix S8).

### Subgroup analyses

Gender stratification was performed for current tobacco use studies (*n* = 6). These analyses repeated the patterns observed in the primary analysis, with similar effect sizes and persistent heterogeneity across the men and women subgroups (Appendix S8). Current cannabis use and CUD could not be stratified by gender owing to insufficient data.

Comparison population stratification (students or employed) was performed for current tobacco use studies (*n* = 10) and cannabis use (*n* = 6). No significant association was observed for current tobacco use among young people with NEET status compared with employed youth (OR = 1.10, 95% CI = 0.91–1.34, *I* ^2^ = 92%), while a significant association with reduced heterogeneity was found when compared with students (OR = 3.05, 95% CI = 2.31–4.03, *I* ^2^ = 82%). For cannabis use, results remained significant for both comparison groups—for students (OR = 1.81, 95% CI = 1.34–2.44, *I* ^2^ = 42%) and for the employed (OR = 1.67, 95% CI = 1.21–2.31, *I* ^2^ = 41%)—with lower heterogeneity in the comparison between young people with NEET status and employed youth. CUD could not be stratified by comparison group owing to insufficient data.

A combined analysis including both current cannabis use and CUD categories was conducted. No significant differences were observed (Appendix S8).

## DISCUSSION

The meta‐analyses on tobacco and cannabis use highlighted significant associations between NEET status and the consumption of each product. However, no significant association was found for CUD. Subgroup analyses stratified by comparison group showed that young people with NEET status have significantly higher current tobacco use compared with students, but no significant difference when compared with employed youth. The observed variation in the prevalence of substance use across NEET profiles, ranging from 27% to 56% for current tobacco use, underscores the importance of not considering NEET youth as a homogenous population, with important implications for prevention or care interventions.

The main findings, showing elevated substance use among NEET individuals, align with previous evidence and support the hypothesis of an association between NEET status and substance use, with substance use acting either as a cause or as a consequence of NEET status [19, 20]. These findings should, however, be interpreted in light of the broader policy context surrounding substance use over the period covered by the included studies. Over the past two decades, several countries in Europe, North America and more broadly across the OECD have implemented increasingly comprehensive tobacco control policies [40], rooted in the WHO Framework Convention on Tobacco Control adopted in 2003 [41], including raising the minimum sales age [42, 43, 44], smoking bans in public places, restrictions on advertising and increased taxation on tobacco products [45, 46, 47]. These policies may have contributed to a significant decline in tobacco use among adolescents and young adults in many high‐income countries. However, the literature highlights that this overall decrease has not been evenly distributed across social groups. Socio‐economic inequalities in smoking persist, and in some cases have widened, with young people from more disadvantaged backgrounds remaining more likely to use tobacco than their more advantaged peers [48, 49, 50]. Our findings corroborate this trend: NEET individuals exhibit significantly higher tobacco use than their non‐NEET counterparts, highlighting these inequalities and the need for tailored smoking prevention and cessation interventions. In addition, recent policy developments regarding cannabis, such as legalisation or decriminalisation in some countries, may also have influenced the patterns of substance use among young people; however, current evidence does not consistently indicate a clear effect on youth cannabis consumption [51, 52, 53].

Taken together, these elements highlight the importance of considering both the socio‐economic context and the policy environment when interpreting trends in substance use among young people.

At the individual level, young people with NEET status face multiple stressors, including economic precarity, uncertainty about their future, and social stigmatisation, which may increase their susceptibility to substance use. Research demonstrates that youth with NEET status are more likely to experience anxiety disorders, behavioural problems or depression [25, 54]. In this context, substance use, especially of cannabis, may serve as a maladaptive coping strategy for managing daily stress, escaping problems or regulating emotions [55, 56]. On the other hand, cannabis use during adolescence and early adulthood has been identified as a risk factor for developing psychiatric disorders [57]. Structural factors further compound these risks, as youth with NEET status often experience limited access to healthcare services and social support networks [21, 58, 59, 60]. Given the higher prevalence of substance use and mental health issues among young people with NEET status compared with their employed or student peers, and considering these access barriers, it highlights a significant public health challenge for healthcare systems and policymakers.

Our quantitative findings lack information on the motivations and context of use among youth with NEET status. Understanding motives for consumption is essential to consider prevention perspectives. According to the Marijuana Motives Measure (MMM) developed by Simons *et al*., cannabis use responds to specific needs, resulting in five types of motivations for consumption: coping (managing negative emotions); social (enhancing social interactions); conformity (peer pressure and integration); enhancement (seeking positive emotions); and expansion (of the mind) [61]. Cannabis use patterns vary according to the underlying motivations, with coping‐motivated use typically occurring in more solitary contexts [62, 63, 64]. This may be particularly prevalent in NEET youth, who experience greater mental health challenges than their employed or student peers [21, 24]. Solitary consumption is particularly concerning given its specific associated risks: it is linked to stronger coping motives [65] and is prospectively associated with a greater likelihood of developing CUD, even after controlling for quantity and frequency of use [66]. The elevated risk may stem from the absence of social comparison—individuals cannot gauge their consumption against peers, potentially leading to more severe consequences [67].

In parallel, the socio‐economic context also influences risk profiles. Adolescents from families of higher socio‐economic status, despite sometimes having similar consumption rates, generally benefit from social and familial support (family supervision, access to care) that reduces negative consequences compared with youth from disadvantaged backgrounds [68]. Young people with NEET status, who often come from these disadvantaged backgrounds, present increased vulnerability to the deleterious consequences of substance use, regardless of the quantities consumed [69].

For young people with NEET status, these risk factors may be compounded by their documented higher levels of social isolation [70], which could potentially increase the likelihood of solitary use that escapes the usual social control of family and peers. This combination of socio‐economic vulnerability and potential solitary use patterns requires particular attention in prevention and intervention strategies.

This meta‐analysis revealed that young people with NEET status had similar tobacco use rates to those of employed peers, but significantly higher rates than students. These results are consistent with existing literature linking lower educational attainment and socio‐economic status to increased smoking prevalence, both common among young people with NEET status [71, 72, 73]. The lower prevalence of tobacco use among students may reflect multiple protective factors: structured environments with smoking restrictions [74]; greater access to university health services, including prevention initiatives [75]; and, importantly, evolving social norms that increasingly discourage tobacco use within academic settings [76]. A recent longitudinal study demonstrated significant increases in support for tobacco‐free campus policies among university students over time [76], with over 86% of students reporting that smoking is discouraged among their peers in some university contexts.

The absence of significant differences in tobacco use between young people with NEET status and their employed peers could reflect the fact that employed youth may work in blue‐collar or service sector jobs that typically require lower educational qualifications, environments where the prevalence of tobacco use has been reported as higher compared with white‐collar or academic settings [77, 78]. The similar consumption rates, despite different financial capacities, raise questions about access and affordability. While employed youth have regular income to purchase tobacco, young people with NEET status may access tobacco through alternative means, potentially exposing them to less regulated and potentially more toxic products obtained through informal networks [79].

Subgroup analyses by gender did not reveal significant differences in tobacco use among young people with NEET status, though these results warrant cautious interpretation owing to the limited number of available studies. This finding is particularly noteworthy when contrasted with the general population, where gender differences in substance use patterns are well documented. Existing literature highlights that young men have higher tobacco use and overall substance consumption than young women [39, 80]. For young people with NEET status, the absence of significant gender differences may suggest an equalisation of substance use risks across the genders. In fact, unemployed youth of both genders were found to have higher levels of tobacco use compared with same‐age peers [81, 82]. Among young women specifically, prolonged unemployment has been identified as a risk factor for developing tobacco use [83], and unemployment is also associated with smoking persistence and lower cessation rates, partly because of reduced access to social and economic resources compared with young men [84]. These factors have been suggested to intensify the use of tobacco as a coping strategy for managing the material and psychosocial stressors related to their gender [84].

Regarding gender aspects of cannabis use, we were unable to conduct analyses in subgroups owing to a lack of available data. Recent research indicates that cannabis use is positively associated with traditional masculine norms, where regular use among boys is perceived as ‘cool’, while habitual use among girls is described as inappropriate [85, 86, 87]. Girls and women who use cannabis are therefore more likely to experience stigma and discrimination compared with men, often being stereotyped as ‘rebellious’ or ‘irresponsible’ [88]. Pregnant women or mothers, a subgroup also present among young people with NEET status, may be particularly affected by gendered discrimination related to substance use according to the broader literature. Evidence from general population studies suggests that care provision for this group is frequently oriented towards foetal or child health, which tends to reduce women to their reproductive roles instead of acknowledging their own health and rights as equally central, particularly in relation to the adverse effects of substance use [86, 89]. However, whether these dynamics apply specifically to NEET young women with family responsibilities remains to be examined empirically.

Age also represents a critical factor in understanding tobacco and cannabis use among young people with NEET status. The NEET definition spans a broad age range [7, 15, 16, 17, 18, 19, 20, 21, 22, 23, 24, 25, 26, 27, 28], encompassing individuals facing very different developmental and social circumstances. Under the age of 25 years, individuals are still undergoing brain development, and substance use during this period has been shown to be especially detrimental, with an increased risk of dependence [90, 91]. Conversely, individuals aged over 25 years and those experiencing prolonged NEET status face elevated rates of anxiety and depression symptoms, and longitudinal evidence suggests that this is associated with a higher likelihood of persistent NEET status over time [24, 92]. Although accounting for age seems, therefore, crucial to understanding the differential impact of consumption patterns and designing tailored interventions, meta‐analyses conducted within this study faced significant limitations in examining age‐specific trends, as many of the included studies used broad age categories (e.g. 10‐year spans) and did not provide substance use data disaggregated by age. Future research should explore age‐specific patterns and the transition from experimentation to regular use among young people with NEET status to inform developmentally appropriate interventions.

### Comparison with existing literature

Compared with prior reviews [25, 26], this meta‐analysis advances the evidence base in three key ways. First, we broadened the inclusion criteria beyond studies explicitly using the term ‘NEET’ to capture research examining youth populations meeting NEET criteria, even when not labelled as such. This approach provides a more comprehensive synthesis of the literature and reduces the risk of excluding relevant studies based solely on terminology. Second, unlike prior reviews that combined all non‐NEET youth into a single comparison group, we distinguished between different reference populations (students vs employed youth). Our subgroup analyses demonstrate that effect sizes differ meaningfully depending on the comparison groups. Third, we established thresholds in substance use frequency and impact, offering homogeneous patterns and allowing a more precise interpretation of risk.

### Strengths and limitations

This meta‐analysis provides the first comprehensive examination of both tobacco and cannabis use across young people with NEET status. While a previous systematic review has examined cannabis use in this population [25], to our knowledge, no study has specifically investigated the association between tobacco use and NEET status or considered the different NEET profiles categorised by Eurofound. Key strengths include a rigorous methodology that followed PRISMA and MOOSE guidelines, a comprehensive search strategy across multiple databases that encompassed both explicit NEET terminology and related population descriptors, the inclusion of 25 studies involving over 90 000 participants, and robust primary and sensitivity analyses.

However, some limitations must be acknowledged. The substantial heterogeneity observed across studies (with *I* ^2^ ranging from 53% to 97%) reflects important methodological differences, varying population characteristics and diverse geographical contexts that limit the generalisability of the pooled estimates. Our reliance on crude odds ratios, owing to heterogeneous adjustment factors across studies, prevented controlling for potential confounding variables and limited our ability to isolate independent associations between NEET status and substance use. Additional methodological concerns include potential measurement bias arising from self‐reported assessments or questionnaires that focus solely on quantity, without accounting for patterns of substance use. A further limitation is related to polysubstance use, which remains insufficiently explored in the literature. In particular, the concurrent use of tobacco and cannabis is relevant for youth, as these substances frequently co‐occur and may amplify risks beyond those associated with single‐substance use. [9, 10, 93].

Furthermore, studies examining young people with NEET status generally provide limited information on group composition, failing to capture the diversity of existing profiles and thereby constraining our understanding of consumption dynamics within this heterogeneous population. Although our search strategy captured NEET youth and the subgroups defined by Eurofound, the available data did not allow us to examine how substance use risk may vary within the NEET population according to additional socio‐demographic factors, such as gender, educational attainment or migratory background. This limitation also raises concerns about selection bias, particularly the under‐representation of certain subgroups, such as homemakers, as well as cross‐cutting groups identified by Eurofound, including migrants and people experiencing homelessness. Insufficient data also prevented comprehensive analyses of CUD (only three studies), gender‐stratified analyses for cannabis outcomes, emergent substances (e.g. e‐cigarettes or nitrous oxide) and an examination of polysubstance use patterns.

Although other smoked substances exist and could theoretically be measured using similar frequency indicators, cannabis and tobacco were the only substances with sufficient published studies to enable meaningful meta‐analysis. This choice has implications for interpretation: our findings cannot be generalized to other smoked substances or substances measured through similar frequency indicators. The distinct profiles of other substances, their varying patterns of use among NEET youth and potential differences in associated risk factors remain areas for future research.

Beyond issues related to group composition, this review primarily examines the prevalence of substance use among young people with NEET status, with limited consideration of the underlying mediating and moderating mechanisms that may help explain these associations. Only one included study conducted mediation analyses, suggesting that NEET status may mediate the relationship between adverse childhood experiences and high levels of tobacco use [94]. Existing literature suggests that early‐life contextual factors, such as low socio‐economic status [30], may operate as moderators in the relationship between unemployment and substance use, while psychosocial factors, including emotional isolation, may act as mediators [95].

Despite these limitations, this meta‐analysis provides robust evidence for targeted prevention and intervention strategies. One research perspective would be to assess the various dynamics of consumption through more in‐depth qualitative studies focusing on NEET populations and specific contexts.

From a clinical and public health perspective, these findings highlight the importance of specifically targeting young people with NEET status within broader youth support frameworks. Universal screening and preventive interventions for substance use should be integrated into NEET‐specific services, including employment support programmes, educational reintegration services and integrated youth services, as part of comprehensive care approaches. Implementing these interventions requires a more nuanced understanding of consumption patterns, considering the diversity of NEET profiles (e.g. short‐term unemployed, long‐term unemployed, re‐entrants, unavailable youth), as well as the direct involvement of youths concerned, to design more relevant and effective actions tailored to the specific needs and circumstances of each NEET subgroup. Given the complex interplay between NEET status, social circumstances and health behaviours, interventions should adopt holistic strategies that simultaneously address socio‐economic barriers, educational opportunities and health needs specific to the challenges faced by young people not in education, employment or training.

## CONCLUSION

This systematic review and meta‐analyses highlight significantly higher levels of tobacco and cannabis use among young people with NEET status compared with their peers of the same age group, particularly when compared with students. The substantial variation in substance use prevalence across different NEET profiles underscores that youth with NEET status represent a heterogeneous population requiring differentiated prevention approaches rather than one‐size‐fits‐all interventions. This represents a significant public health concern, given the socio‐demographic vulnerabilities to which this population is exposed, coupled with their documented higher levels of social isolation and limited access to healthcare services and support networks. Prevention should be considered part of a comprehensive and integrated approach to health, taking into account individual factors as well as the collective and environmental factors that can be addressed.

## AUTHOR CONTRIBUTIONS

**Clara Eyraud:** Conceptualization; data curation; formal analysis; visualization; software; methodology; funding acquisition; investigation; writing—original draft; writing—review and editing. **Claire Collin:** Conceptualization; data curation; formal analysis; investigation; methodology; writing—review and editing. **Philippe Martin:** Conceptualization; funding acquisition; methodology; supervision; validation; writing—review and editing. **Corinne Alberti:** Validation; supervision; writing—review and editing. **Laëtitia Minary:** Validation; writing—review and editing; supervision. **Agnès Dumas:** Data curation; supervision; validation; writing—review and editing. **Enora Le Roux:** Conceptualization; methodology; data curation; formal analysis; funding acquisition; investigation; software; supervision; validation; visualization; writing—review and editing.

## DECLARATION OF INTERESTS

None.

## STUDY REGISTRATION

The study protocol has previously been registered in PROSPERO (CRD42023401994).

## Supporting information


**Appendix S1.** PRISMA and MOOSE checklist.
**Appendix S2.** Search strategy.
**Appendix S3.** Tables of quality assessment.
**Appendix S4.** GRADE.
**Appendix S5.** Characteristics of the articles.
**Appendix S6.** Age.
**Appendix S7.** NEET subgroup descriptions.
**Appendix S8.** Sensitivity and subgroup analyses.

## Data Availability

The data that support the findings of this study are available from the corresponding author upon reasonable request.
