# Supplementary material for: Tobacco and cannabis use in the young ‘Not in Employment, Education or Training’ population: A systematic review and meta‐analysis
Source: Addiction. 2026 Jun 1;121(10):2591–606. doi: 10.1111/add.70477 (PMC13578992; doi:10.1111/add.70477)
Supplement: Supplementary file 1 [file ADD-121-2591-s001.docx]

# SUPPORTING INFORMATION

## Appendix A – PRISMA and MOOSE Checklist

### Table A.1 - PRISMA checklist for reporting systematic reviews

| **Section and Topic** | **Item #** | **Checklist item** | **Page** |
| --- | --- | --- | --- |
| **TITLE** | | |  |
| Title | 1 | Identify the report as a systematic review. | 1 |
| **ABSTRACT** | | |  |
| Abstract | 2 | See the PRISMA 2020 for Abstracts checklist. | Appendix 1 |
| **INTRODUCTION** | | |  |
| Rationale | 3 | Describe the rationale for the review in the context of existing knowledge. | 4-5 |
| Objectives | 4 | Provide an explicit statement of the objective(s) or question(s) the review addresses. | 5 |
| **METHODS** | | |  |
| Eligibility criteria | 5 | Specify the inclusion and exclusion criteria for the review and how studies were grouped for the syntheses. | 6 |
| Information sources | 6 | Specify all databases, registers, websites, organisations, reference lists and other sources searched or consulted to identify studies. Specify the date when each source was last searched or consulted. | 6 |
| Search strategy | 7 | Present the full search strategies for all databases, registers and websites, including any filters and limits used. | 6 |
| Selection process | 8 | Specify the methods used to decide whether a study met the inclusion criteria of the review, including how many reviewers screened each record and each report retrieved, whether they worked independently, and if applicable, details of automation tools used in the process. | 6 - 7 |
| Data collection process | 9 | Specify the methods used to collect data from reports, including how many reviewers collected data from each report, whether they worked independently, any processes for obtaining or confirming data from study investigators, and if applicable, details of automation tools used in the process. | 7 |
| Data items | 10a | List and define all outcomes for which data were sought. Specify whether all results that were compatible with each outcome domain in each study were sought (e.g. for all measures, time points, analyses), and if not, the methods used to decide which results to collect. | 7 |
|  | 10b | List and define all other variables for which data were sought (e.g. participant and intervention characteristics, funding sources). Describe any assumptions made about any missing or unclear information. | 7 |
| Study risk of bias assessment | 11 | Specify the methods used to assess risk of bias in the included studies, including details of the tool(s) used, how many reviewers assessed each study and whether they worked independently, and if applicable, details of automation tools used in the process. | 7 |
| Effect measures | 12 | Specify for each outcome the effect measure(s) (e.g. risk ratio, mean difference) used in the synthesis or presentation of results. | NA |
| Synthesis methods | 13a | Describe the processes used to decide which studies were eligible for each synthesis (e.g. tabulating the study intervention characteristics and comparing against the planned groups for each synthesis (item #5)). | NA |
|  | 13b | Describe any methods required to prepare the data for presentation or synthesis, such as handling of missing summary statistics, or data conversions. | 7 - 8 |
|  | 13c | Describe any methods used to tabulate or visually display results of individual studies and syntheses. | NA |
|  | 13d | Describe any methods used to synthesize results and provide a rationale for the choice(s). If meta-analysis was performed, describe the model(s), method(s) to identify the presence and extent of statistical heterogeneity, and software package(s) used. | 8-9 |
|  | 13e | Describe any methods used to explore possible causes of heterogeneity among study results (e.g. subgroup analysis, meta-regression). | 9 |
|  | 13f | Describe any sensitivity analyses conducted to assess robustness of the synthesized results. | 9 |
| Reporting bias assessment | 14 | Describe any methods used to assess risk of bias due to missing results in a synthesis (arising from reporting biases). | 7 |
| Certainty assessment | 15 | Describe any methods used to assess certainty (or confidence) in the body of evidence for an outcome. | NA |
| **RESULTS** | | |  |
| Study selection | 16a | Describe the results of the search and selection process, from the number of records identified in the search to the number of studies included in the review, ideally using a flow diagram. | Figure 1 |
|  | 16b | Cite studies that might appear to meet the inclusion criteria, but which were excluded, and explain why they were excluded. | Figure 1 |
| Study characteristics | 17 | Cite each included study and present its characteristics. | Appendix 4 |
| Risk of bias in studies | 18 | Present assessments of risk of bias for each included study. | Appendix 3 |
| Results of individual studies | 19 | For all outcomes, present, for each study: (a) summary statistics for each group (where appropriate) and (b) an effect estimate and its precision (e.g. confidence/credible interval), ideally using structured tables or plots. | Table 1 and Table 2 |
| Results of syntheses | 20a | For each synthesis, briefly summarise the characteristics and risk of bias among contributing studies. | Table 1 and appendix 4 |
|  | 20b | Present results of all statistical syntheses conducted. If meta-analysis was done, present for each the summary estimate and its precision (e.g. confidence/credible interval) and measures of statistical heterogeneity. If comparing groups, describe the direction of the effect. | 14 -17 |
|  | 20c | Present results of all investigations of possible causes of heterogeneity among study results. | 17 - 18 |
|  | 20d | Present results of all sensitivity analyses conducted to assess the robustness of the synthesized results. | 17 - 18 |
| Reporting biases | 21 | Present assessments of risk of bias due to missing results (arising from reporting biases) for each synthesis assessed. | NA |
| Certainty of evidence | 22 | Present assessments of certainty (or confidence) in the body of evidence for each outcome assessed. | NA |
| **DISCUSSION** | | |  |
| Discussion | 23a | Provide a general interpretation of the results in the context of other evidence. | 18 |
|  | 23b | Discuss any limitations of the evidence included in the review. | 21-22 |
|  | 23c | Discuss any limitations of the review processes used. | 18-21 |
|  | 23d | Discuss implications of the results for practice, policy, and future research. | 18-21 |
| **OTHER INFORMATION** | | |  |
| Registration and protocol | 24a | Provide registration information for the review, including register name and registration number, or state that the review was not registered. | 25 |
|  | 24b | Indicate where the review protocol can be accessed, or state that a protocol was not prepared. | NA |
|  | 24c | Describe and explain any amendments to information provided at registration or in the protocol. | NA |
| Support | 25 | Describe sources of financial or non-financial support for the review, and the role of the funders or sponsors in the review. | 25 |
| Competing interests | 26 | Declare any competing interests of review authors. | 25 |
| Availability of data, code and other materials | 27 | Report which of the following are publicly available and where they can be found: template data collection forms; data extracted from included studies; data used for all analyses; analytic code; any other materials used in the review. | Supporting Information |

*From:*  Page MJ, McKenzie JE, Bossuyt PM, Boutron I, Hoffmann TC, Mulrow CD, et al. The PRISMA 2020 statement: an updated guideline for reporting systematic reviews. BMJ 2021;372:n71. doi: 10.1136/bmj.n71

Table A.2 - MOOSE checklist for reporting meta-analyses

| **Reporting Criteria** | Page |
| --- | --- |
| **Reporting of Background** |  |
| Problem definition | 4-5 |
| Hypothesis statement | 4-5 |
| Description of Study Outcome(s) | 4-5 |
| Type of exposure or intervention used | 4-5 |
| Type of study design used | NA |
| Study population | 4-5 |
| **Reporting of Search Strategy** |  |
| Qualifications of searchers (eg, librarians and investigators) | 6 |
| Search strategy, including time period included in the synthesis and keywords | 6 |
| Effort to include all available studies, including contact with authors | 8-9 |
| Databases and registries searched | 6 |
| Search software used, name and version, including special features used (eg, explosion | 7 |
| Use of hand searching (eg, reference lists of obtained articles) | NA |
| List of citations located and those excluded, including justification | Figure 1 |
| Method for addressing articles published in languages other than English | NA |
| Method of handling abstracts and unpublished studies | Figure 1 |
| Description of any contact with authors | NA |
| **Reporting of Methods** |  |
| Description of relevance or appropriateness of studies assembled for assessing the hypothesis to be tested | 6-8 |
| Rationale for the selection and coding of data (eg, sound clinical principles or convenience) | 7-8 |
| Documentation of how data were classified and coded (eg, multiple raters, blinding, and interrater reliability) | 7-8 |
| Assessment of confounding (eg, comparability of cases and controls in studies where appropriate | NA |
| **Reporting Criteria** |  |
| Assessment of study quality, including blinding of quality assessors; stratification or regression on possible predictors of study results | Appendix 3 |
| Assessment of heterogeneity | 8-9 |
| Description of statistical methods (eg, complete description of fixed or random effects models, justification of whether the chosen models account for predictors of study results, dose-response models, or cumulative meta-analysis) in sufficient detail to be replicated | 8-9 |
| Provision of appropriate tables and graphics | 10 -18 |
| **Reporting of Results** |  |
| Table giving descriptive information for each study included | NA |
| Results of sensitivity testing (eg, subgroup analysis) | Appendix 6 |
| Indication of statistical uncertainty of findings | 10-17 |
| **Reporting of Discussion** |  |
| Quantitative assessment of bias (eg, publication bias) | 18-22 |
| Justification for exclusion (eg, exclusion of non–English-language citations) | Figure1 |
| Assessment of quality of included studies | Appendix 3 |
| **Reporting of Conclusions** |  |
| Consideration of alternative explanations for observed results | 18-22 |
| Generalization of the conclusions (ie, appropriate for the data presented and within the domain of the literature review) | 18- 22 |
| Guidelines for future research | 22 |
| Disclosure of funding source | 24 |

## *From: Stroup, D.F., Berlin, J.A., Morton, S.C., Olkin, I., Williamson, G.D., Rennie, D., Moher, D., Becker, B.J., Sipe, T.A., and Thacker, S.B. (2000) Meta-analysis of observational studies in epidemiology: a proposal for reporting. Meta-analysis Of Observational Studies in Epidemiology (MOOSE) group. JAMA, 283 (15), 2008–2012* Appendix B - Search strategy

**PubMed search strategy**

**Timeline filter: 1999 – 2025**

### **Algorithm:**

("young adult"[MeSH Terms] OR "young adult*"[Text Word] OR "adolescent" [MeSH Terms] OR "adolescent*" [Text Word] OR "young"[Text Word] OR "youth" [Text Word] OR "aya" [Text Word]OR “teen*”[Text Word])

AND

("neet" [Text Word] OR "not in employment, education or training" [All Fields] OR "nor in employment, education or training" [All Fields] OR ("employment"[Text Word] AND "not in"[Text Word]) OR "not in employment" [All Fields] OR "not in education" [Text Word] OR "not in training" [Text Word] OR "neither in employment nor in education or training" [Text Word] OR "unavailable*" [Text Word] OR "disengaged" [Text Word] OR “voluntary neet”[Text Word] OR “opportunity-seekers”[All Fields] OR "unemployment"[MeSH Terms] OR "unemploym*" [Text Word] OR "student dropouts"[MeSH Terms] OR "student dropout*" [Text Word]OR "jobless"[Text Word] OR "Disabled Persons"[Mesh Terms] OR "disabled person*"[Text Word] OR "young carer*"[Text Word] OR "unoccupied*"[Text Word] OR "not in school"[Text Word] OR "inactive youth"[Text Word] OR "academically disengag*"[All Fields] OR "vocationally disengag*"[All Fields] OR "social isolation"[MeSH Terms] OR "social exclusion*"[Text Word] OR “seeking opportunit*”[Text Word] OR “job-seek*”[Text Word] OR “seek opportunit*”[Text Word] OR “gap year*”[Text Word])

NOT

("protein"[Text Word] OR "proteic"[Text Word])

AND

(("substance-related disorders"[MeSH Terms] OR "substance-related disorder*"[Text Word] OR "drug users"[MeSH Terms] OR "drug user*"[Text Word] OR "alcoholics"[MeSH Terms] OR "alcoholic*"[Text Word] OR "underage drinking"[MeSH Terms] OR "underage drinking"[Text Word] OR "binge drinking"[MeSH Terms] OR "binge drinking"[Text Word] OR "smokers"[MeSH Terms] OR "smoker" [Text Word] OR "smoking"[MeSH Terms ] OR "smoking"[Text Word] OR "tobacco use"[MeSH Terms] OR "tobacco use*"[Text Word])

OR

(("substance*"[Text Word]OR"psychoactive drug*"[Text Word]OR"psychoactive substance*"[Text Word]OR "drug*"[Text Word]OR"alcohol"[Text Word]OR"tobacco"[Text Word]OR "cannabis"[Text Word]OR "marijuana"[Text Word]OR "cocaine"[Text Word]OR "amphetamine"[Text Word]OR "heroin"[Text Word]OR “opioid”[Text Word]OR “medication”[Text Word]OR “ecstasy”[Text Word]OR “mdma”[Text Word]OR “class A drug”[Text Word] OR “class B drug”[Text Word] OR “class C drug”[Text Word])

AND

("use"[Text Word] OR "abuse"[Text Word] OR" addiction*"[Text Word] OR "misuse"[Text Word] OR "dependenc*"[Text Word] OR"user*" [Text Word])))

**Cairn**

(«jeunes» OU «adolescent» ou «jeunes adultes»)ET(«NEET» OU «not in employment education or training» OU «neither in employment nor in education or training» OU «nor in employment, education or training» OU «ni en emploi, ni en étude, ni en formation» OU «jeunes déscolarisés sans emploi» OU «jeunes au chômage» OU «jeunes déscolarisé» OU «jeunes désocialisé» ) ET («addiction» OU «substance psychoactive» OU «consommation de substance» OU «dépendance» OU «toxicomane» OU «conduite à risque»)

**PsychInfo**

(youth or adolescents or young people or teen or young adults)

AND

(neet or not in education employment or training or neither in education employment or training)

AND

(substance abuse or substance use or drug abuse or drug addiction or drug use)

**Web of Science**

(“young adult” OR “adolescent” OR “young” OR “youth” OR “aya” OR “teen”)

AND

(“neet” OR “not in employment education or training” OR “nor in employment education or training” OR “neither in employment nor in education or training” OR “unavailable” OR “disengaged” OR “voluntary neet” OR “opportunity-seekers” OR “unemployment” OR “student dropouts” OR “jobless” OR “disabled person” OR “young carer” OR “unoccupied” OR “not in school” OR “inactive youth” OR “academically disengaged” OR “vocationally disengaged” OR “social isolation” OR “social exclusion” OR “seeking opportunity” OR “job-seeker” OR “seek opportunity” OR “gap year”)

NOT

(“protein” OR “proteic”)

AND

((“smoker” OR “smoking” OR “tobacco use”) OR ((“tobacco” OR “cannabis” OR “marijuana”) AND (“use” OR “abuse” OR “addiction” OR “misuse” OR “dependence” OR “user”)))

## Appendix C - Tables of quality assessment

Table C.1 - Quality assessment of transversal studies

| **Articles** | Objective description | Study design appropriate | Comparison group | Subject description | Intervention and random allocation description | Intervention and blinding of investigators description | Intervention and blinding of subjects description | Outcome and exposure description | Sample size appropriate | Analytic methods description | Estimate of variance reported | Controlled for confounding | Results reported in sufficient detail | Conclusions supported by the results | **Article score** | **Total score** | **Articles score/ Total score** | **Result** |
| --- | --- | --- | --- | --- | --- | --- | --- | --- | --- | --- | --- | --- | --- | --- | --- | --- | --- | --- |
| **CH. Stewart 2017** | 2 | 2 | 2 | 0 | NA | NA | NA | 1 | 2 | 2 | 2 | 2 | 0 | 2 | 17 | 22 | 0.77 | STRONG |
| **D. Lawrence 2006** | 2 | 1 | 2 | 2 | NA | NA | NA | 2 | 2 | 2 | 2 | 2 | 2 | 2 | 21 | 22 | 0.95 | STRONG |
| **N. Neault 2012** | 2 | 1 | 2 | 1 | NA | NA | NA | 1 | 0 | 1 | 1 | NA | 1 | 2 | 12 | 20 | 0.60 | MODERATE |
| **D. Karaoglan 2022** | 2 | 1 | 1 | 1 | NA | NA | NA | 1 | 2 | 1 | 2 | 2 | 1 | 0 | 14 | 22 | 0.64 | MODERATE |
| **C. Tanton 2021** | 2 | 2 | 2 | 1 | NA | NA | NA | 1 | 2 | 1 | 2 | 1 | 2 | 2 | 18 | 22 | 0.82 | STRONG |
| **M. Basta 2019** | 2 | 2 | 1 | 1 | NA | NA | NA | 1 | 1 | 2 | 2 | 2 | 1 | 2 | 17 | 22 | 0.77 | STRONG |
| **JA. De la Torre** | 2 | 2 | 2 | 1 | NA | NA | NA | 1 | 2 | 1 | 2 | 2 | 2 | 2 | 18 | 22 | 0.82 | STRONG |
| **ML. Steele 2021** | 2 | 2 | 2 | 1 | NA | NA | NA | 1 | 1 | 2 | 2 | 2 | 1 | 2 | 17 | 22 | 0.77 | STRONG |
| **M. Hwang 2012** | 2 | 2 | 1 | 1 | NA | NA | NA | 1 | 1 | 1 | 2 | 1 | 1 | 2 | 15 | 22 | 0.68 | MODERATE |
| **T. H Stea 2019** | 2 | 1 | 2 | 1 | NA | NA | NA | 1 | 1 | 2 | 2 | 2 | 1 | 2 | 17 | 22 | 0.77 | STRONG |
| **S. Legleye 2008** | 1 | 2 | 2 | 1 | NA | NA | NA | 1 | 2 | 1 | 2 | 2 | 1 | 2 | 17 | 22 | 0.77 | STRONG |
| **I. Aguilar-Palacio 2015** | 2 | 2 | 2 | 1 | NA | NA | NA | 1 | 2 | 1 | 2 | 2 | 1 | 2 | 18 | 22 | 0.82 | STRONG |
| **AJ. Caban-Martinez 2012** | 2 | 2 | 2 | 1 | NA | NA | NA | 1 | 2 | 1 | 2 | NA | 1 | 2 | 16 | 20 | 0.80 | STRONG |
| **CX. Gao 2023** | 2 | 2 | 2 | 1 | NA | NA | NA | 1 | 2 | NA | NA | NA | 1 | 2 | 13 | 16 | 0.81 | STRONG |

*From: Kmet LM. Cook LS. Lee RC. Standard Quality Assessment Criteria for Evaluating Primary Research Papers from a Variety of Fields. 2004. Available from:* [*https://doi.org/10.7939/R37M04F16*](https://doi.org/10.7939/R37M04F16)

Table C.2 - Quality assessment of cohort studies

| **Article** | **Selection** | | | | | **Comparability** | | **Outcome** | | | | **Score** | **Result** |
| --- | --- | --- | --- | --- | --- | --- | --- | --- | --- | --- | --- | --- | --- |
| **L. Rodwell 2018** | * | * | 0 | * | 3 | ** | 2 | 0 | * | * | 2 | 7 | good |
| **T. Tabuchi 2018** | * | * | * | * | 4 | 0 * | 1 | 0 | * | * | 2 | 7 | good |
| **I. Reine 2004** | * | * | 0 | * | 3 | ** | 2 | 0 | * | * | 2 | 7 | good |
| **AM. Arria 2012** | * | * | * | * | 4 | ** | 2 | 0 | * | * | 2 | 8 | good |
| **S. Goldman-Mellor** | * | * | 0 | * | 3 | ** | 2 | 0 | * | * | 2 | 7 | good |
| **L. Shananhan 2021** | * | * | 0 | * | 3 | ** | 2 | 0 | * | * | 2 | 7 | good |
| **S. Baggio 2015** | * | * | 0 | 0 | 2 | ** | 2 | 0 | * | * | 2 | 6 | fair |
| **R. El Haddad 2023** | * | * | 0 | * | 3 | ** | 2 | 0 | * | * | 2 | 7 | good |
| **B. Redonnet 2012** | * | * | 0 | * | 3 | ** | 2 | 0 | * | * | 2 | 7 | good |
| **WH. Lin 2021** | * | * | 0 | * | 3 | ** | 2 | 0 | * | * | 2 | 7 | good |
| **J. Homel 2014** | * | * | * | * | 4 | 0* | 1 | 0 | * | * | 2 | 7 | good |

*From: Wells G. Shea B. O’Connell D. Peterson J. Welch V. Losos M. et al. The Newcastle-Ottawa Scale (NOS) for assessing the quality of nonrandomised studies in meta-analyses. 2013. Available from:* [*http://www.ohri.ca/programs/clinical_epidemiology/oxford.asp*](http://www.ohri.ca/programs/clinical_epidemiology/oxford.asp)

## Appendix D – GRADE

Table D - GRADE assessment

| **Outcome** | **Number of studies** | **OR [CI 95%]** | **Design** | **Risk of bias** | **Imprecision** | **Inconsistency** | **Indirectness** | **Publication bias** | **Other considerations** | **Quality of evidence** |
| --- | --- | --- | --- | --- | --- | --- | --- | --- | --- | --- |
| Current smoker | 18 | 1.92 [1.46-2.58] | Observational studies | Not serious | Serious | Serious | Not serious | Serious | None | Very low |
| Cannabis use | 9 | 2.14 [1.68-2.36] | Observational studies | Not serious | Serious | Serious | Not serious | Serious | None | Very low |
| Cannabis use disorder | 3 | 2.89 [0.42-19.91] | Observational studies | Not serious | Serious | Serious | Not serious | Serious | None | Very low |

OR: Odds Ratio. These are the pooled ORs derived from the meta-analyses in the article.

CI: Confident Interval

**Appendix E - Characteristics of the articles**

Table E - Characteristics of the articles

| **Date** | **Country** | **Study Design** | **Type of substance use** | **Article** | **Age of**  **total population** | **Mean Age** | **SD** | **Population of interest (N)** | | | **Men (N)** | **Women (N)** | **Comparison population (N)** | | **Men (N)** | **Women (N)** | **OR [CI 95%]** | **Evaluation tool** | **Quality Assessment** |
| --- | --- | --- | --- | --- | --- | --- | --- | --- | --- | --- | --- | --- | --- | --- | --- | --- | --- | --- | --- |
| 2015 | Switzerland | Cohort | Current smoker | S. Baggio | 17 - 28 | 20 | 1.2 | NEET | (N=292) | | 292 | 0 | Not NEET | (N=4,466) | 292 | 0 | 1.02†  [0.59 ; 1.78] | NOS | Fair |
| 2016 | England and Wales | Cohort | Current smoker | S. Goldman-Mellor | 18 | NA | NA | NEET | (N=239) | | 108 | 131 | Not NEET | (N=1,827) | 108 | 131 | 1.45  [1.08 ; 1.96] | NOS | Good |
| 2017 | England And Scotland | Transversal | Current smoker | CH. Stewart | 16 - 24 | 20.2† | NA | NEET | (N=715†) | | 263 | 478 | Not NEET | (N=3,557†) | 263 | 478 | 0.73  [0.59 ; 0.90] | QualSyst | Strong |
| 2019 | Greece | Transversal | Current smoker | M. Basta | 15 - 24 | 21‡ | 18–23‡ | NEET | (N=450) | | 216 | 234 | Not NEET | (N=2,291) | 216 | 234 | 0.96†  [0.66 ; 1.41] | QualSyst | Strong |
| 2019 | Norway | Transversal | Current smoker | T.H. Stea | 16 - 21 | NA | NA | NEET | (N=96) | | 53 | 43 | Student | (N=384) | 212 | 172 | 2.41†  [1.50 ; 3.88] | QualSyst | Strong |
| 2021 | Australia | Transversal | Current smoker | ML. Steele | 14 - 17 | 16 | 1 | NEET | (N=120) | | NA | NA | Not NEET | (N=341) | NA | NA | 2.05†  [1.35 ; 3.13] | QualSyst | Strong |
| 2021 | United Kingdom | Transversal | Current smoker | C. Tanton | 16 - 24 | NA | NA | NEET | (N=428†) | | 189 | 239 | Not NEET | (N=2,016†) | 189 | 239 | 0.95†  [0.85 ; 1.50] | QualSyst | Strong |
| 2022 | Turkey | Transversal | Current smoker | D. Karaoglan | 15 - 29 | 21.98 | 4.4 | NEET | (N=3,832†) | | NA | NA | Not NEET | (N=9,701†) | NA | NA | 0.80  [0.72 ; 0.89] | QualSyst | Strong |
| 2022 | Taiwan | Cohort | Current smoker | WH. Lin | 15 - 25 | 22 | 0.49 | NEET | (N=208) | | NA | NA | Not NEET | (N=2,585) | NA | NA | 5.60†  [1.06 ; 29.67] | NOS | Good |
| 2004 | Sweden | Cohort | Current smoker | I. Reine | 21 | NA | NA | Unemployed | (N=166†) | | 93 | 83 | Not NEET | (N=878†) | 464 | 414 | 3.53†  [2.50 ; 4.98] | NOS | Good |
| 2006 | USA | Transversal | Current smoker | D. Lawrence | 18 - 24 | NA | NA | Unemployed & Other | (N=4,705) | | NA | NA | Employed | (N=10,666) | NA | NA | 1.56†  [1.42 ; 1.71] | QualSyst | Strong |
| 2008 | France | Transversal | Current smoker | S. Legleye | 18 - 25 | 21.61† | NA | Unemployed | (N=538) | | 227 | 311 | Not NEET | (N=2,770) | 227 | 311 | 1.00  [0.81 ; 1.24] | QualSyst | Strong |
| 2012 | France | Cohort | Current smoker | B. Redonnet | 22 - 35 | NA | NA | Unemployed | (N=81†) | | 37 | 44 | Not NEET | (N=1,002†) | 37 | 44 | 1.55†  [0.87 ; 2.76] | QualSyst | Moderate |
| 2012 | USA | Transversal | Current smoker | M. Hwang | 21 - 25 | 23.4 | 0.9 | Unemployed | (N=139†) | | NA | NA | Employed | (N=76†) | NA | NA | 0.43†  [0.22 ; 0.84] | QualSyst | Moderate |
| 2012 | USA | Transversal | Current smoker | AJ. Caban-Martinez | 18 - 24 | NA | NA | Unemployed | (N=10,102) | | 4029 | 6073 | Employed | (N=16,908) | 4029 | 6073 | 1.12†  [1.03 ; 1.22] | QualSyst | Strong |
| 2015 | Spain | Transversal | Current smoker | I. Aguilar-Palacio | 16 - 24 | NA | NA | Unemployed | | (N=447) | 230 | 217 | Not NEET | (N=3,064) | 1531 | 1533 | 1.97†  [1.61 ; 2.41] | QualSyst | Strong |
| 2018 | Japan | Cohort | Current smoker | T. Tabuchi | 17 - 18 | NA | NA | Dropout | | (N=130) | 79 | 51 | Not dropout | (N=565) | 308 | 257 | 7.88†  [5.18 ; 11.99] | NOS | Good |
| 2023 | France | Cohort | Current smoker | R. El Haddad | 18 - 30 | 21.4 | 2.4 | Unemployed & Other | | (N=255) | 90 | 165 | Not NEET | (N=2,618) | 90 | 165 | 0.89†  [0.52 ; 1.54] | NOS | Good |
| 2015 | Switzerland | Cohort | Cannabis use | S. Baggio | 17 - 28 | 20 | 1.2 | NEET | | (N=292) | 292 | NA | Not NEET | (N=4,466) | 292 | NA | 1.08†  [0.80 ; 1.44] | NOS | Fair |
| 2018 | Australia | Cohort | Cannabis use | L. Rodwell | 20 - 21 | 20.7 | NA | NEET | | (N=165) | 77 | 88 | Not NEET | (N=1,773) | 77 | 88 | 1.54†  [1.03 ; 2.31] | NOS | Good |
| 2021 | Switzerland | Cohort | Cannabis use | L. Shananhan | 20 | NA | NA | NEET | | (N=35†) | NA | NA | Not NEET | (N=1,118†) | NA | NA | 2.77  [1.41 ; 5.46] | NOS | Good |
| 2023 | Australia | Transversal | Cannabis use | CX. Gao | 12 - 25 | 18‡ | 16 - 20‡ | NEET | | (N=89) | NA | NA | Not NEET | (N=647) | NA | NA | 2.05†  [1.39 ; 3.03] | QualSyst | Strong |
| 2008 | France | Transversal | Cannabis use | S. Legleye | 18 - 25 | 21.61† | NA | Unemployed | | (N=538) | 227 | 311 | Not NEET | (N=2,770) | 227 | 311 | 1.00  [0.81 ; 1.24] | QualSyst | Strong |
| 2014 | Canada | Cohort | Cannabis use | J. Homel | 15 - 25 | NA | NA | Dropout | | (N=101) | NA | NA | Not dropout | (N=191) | NA | NA | 1.14†  [0.63 ; 5.21] | NOS | Good |
| 2023 | France | Cohort | Cannabis use | R. El Haddad | 18 - 30 | 21.4 | 2.4 | Unemployed & Other | | (N=255) | 90 | 165 | Not NEET | (N=2,618) | 90 | 165 | 0.89†  [0.52 ; 1.54] | NOS | Good |
| 2012 | USA | Cohort | Cannabis use | AM. Arria | 23 - 25 | NA | NA | Unemployed | | (N=34) | 17 | 17 | Employed | (N=510) | 17 | 17 | 1.15†  [0.57 ; 2.31] | NOS | Good |
| 2012 | USA | Transversal | Cannabis use | N. Neault | 18 - 30 | 22.9 | 2.8 | Unemployed | | (N=61) | 61 | 0 | Not NEET | (N=26) | 61 | 0 | 2.72†  [0.90 ; 8.20] | QualSyst | Moderate |
| 2012 | France | Cohort | CUD | B. Redonnet | 22 - 35 | NA | NA | Unemployed | | (N=81†) | 37 | 44 | Not NEET | (N=1,002†) | 37 | 44 | 1.55†  [0.87 ; 2.76] | QualSyst | Moderate |
| 2016 | England and Wales | Cohort | CUD | S. Goldman-Mellor | 18 | NA | NA | NEET | | (N=239) | 108 | 131 | Not NEET | (N=1,827) | 108 | 131 | 1.45  [1.08 ; 1.96] | NOS | Good |
| 2021 | Spain | Transversal | CUD | JA. De la Torre | 16 - 35 | NA | NA | Unemployed | | (N=407) | 316 | 91 | Not NEET | (N=1,172) | 801 | 371 | 1.95†  [1.55 ; 2.46] | QualSyst | Strong |

†Recalculate based on the data from the article; ‡ Median and IQR

CI: Confident Interval; CUD: Cannabis use disorder; NA: Not Available; NEET: Not in Employment, Education or Training; NOS: Newcastle-Ottawa Scale; OR: Odds Ratio; QualSyst: Standard Quality Assessment Criteria; SD: Standard deviation; USA: United States of America

## Appendix F – Age

Table F – Age distribution of populations by article

| **Article** | **Population of interest (N)** | **Age** |  | **15** | **16** | **17** | **18** | **19** | **20** | **21** | **22** | **23** | **24** | **25** | **26** | **27** | **28** | **29** | **30** |  |  |
| --- | --- | --- | --- | --- | --- | --- | --- | --- | --- | --- | --- | --- | --- | --- | --- | --- | --- | --- | --- | --- | --- |
| S. Baggio | NEET | 17 - 28 |  |  |  |  |  |  |  |  |  |  |  |  |  |  |  |  |  |  | **Current tobacco use** |
| S. Goldman-Mellor | NEET | 18 |  |  |  |  |  |  |  |  |  |  |  |  |  |  |  |  |  |  |  |
| CH. Stewart | NEET | 16 - 24 |  |  |  |  |  |  |  |  |  |  |  |  |  |  |  |  |  |  |  |
| M. Basta | NEET | 15 - 24 |  |  |  |  |  |  |  |  |  |  |  |  |  |  |  |  |  |  |  |
| T.H. Stea | NEET | 16 - 21 |  |  |  |  |  |  |  |  |  |  |  |  |  |  |  |  |  |  |  |
| ML. Steele | NEET | 14 - 17 | 14 |  |  |  |  |  |  |  |  |  |  |  |  |  |  |  |  |  |  |
| C. Tanton | NEET | 16 - 24 |  |  |  |  |  |  |  |  |  |  |  |  |  |  |  |  |  |  |  |
| D. Karaoglan | NEET | 15 - 29 |  |  |  |  |  |  |  |  |  |  |  |  |  |  |  |  |  |  |  |
| WH. Lin | NEET | 15 - 25 |  |  |  |  |  |  |  |  |  |  |  |  |  |  |  |  |  |  |  |
| I. Reine | Unemployed | 21 |  |  |  |  |  |  |  |  |  |  |  |  |  |  |  |  |  |  |  |
| D. Lawrence | Unemployed & Other | 18 - 24 |  |  |  |  |  |  |  |  |  |  |  |  |  |  |  |  |  |  |  |
| S. Legleye | Unemployed | 18 - 25 |  |  |  |  |  |  |  |  |  |  |  |  |  |  |  |  |  |  |  |
| B. Redonnet | Unemployed | 22 - 35 |  |  |  |  |  |  |  |  |  |  |  |  |  |  |  |  |  | 35 |  |
| M. Hwang | Unemployed | 21 - 25 |  |  |  |  |  |  |  |  |  |  |  |  |  |  |  |  |  |  |  |
| AJ. Caban-Martinez | Unemployed | 18 - 24 |  |  |  |  |  |  |  |  |  |  |  |  |  |  |  |  |  |  |  |
| I. Aguilar-Palacio | Unemployed | 16 - 24 |  |  |  |  |  |  |  |  |  |  |  |  |  |  |  |  |  |  |  |
| T. Tabuchi | Dropout | 17 - 18 |  |  |  |  |  |  |  |  |  |  |  |  |  |  |  |  |  |  |  |
| R. El Haddad | Unemployed & Other | 18 - 30 |  |  |  |  |  |  |  |  |  |  |  |  |  |  |  |  |  |  |  |
| S. Baggio | NEET | 17 - 28 |  |  |  |  |  |  |  |  |  |  |  |  |  |  |  |  |  |  | **Current cannabis use** |
| L. Rodwell | NEET | 20 - 21 |  |  |  |  |  |  |  |  |  |  |  |  |  |  |  |  |  |  |  |
| L. Shananhan | NEET | 20 |  |  |  |  |  |  |  |  |  |  |  |  |  |  |  |  |  |  |  |
| CX. Gao | NEET | 12 25 | 12 |  |  |  |  |  |  |  |  |  |  |  |  |  |  |  |  |  |  |
| S. Legleye | Unemployed | 18 - 25 |  |  |  |  |  |  |  |  |  |  |  |  |  |  |  |  |  |  |  |
| J. Homel | Dropout | 15 - 25 |  |  |  |  |  |  |  |  |  |  |  |  |  |  |  |  |  |  |  |
| R. El Haddad | Unemployed & Other | 18 - 30 |  |  |  |  |  |  |  |  |  |  |  |  |  |  |  |  |  |  |  |
| AM. Arria | Unemployed | 23 - 25 |  |  |  |  |  |  |  |  |  |  |  |  |  |  |  |  |  |  |  |
| N. Neault | Unemployed | 18 - 30 |  |  |  |  |  |  |  |  |  |  |  |  |  |  |  |  |  |  |  |
| B. Redonnet | Unemployed | 22 - 35 |  |  |  |  |  |  |  |  |  |  |  |  |  |  |  |  |  | 35 | **CUD** |
| S. Goldman-Mellor | NEET | 18 |  |  |  |  |  |  |  |  |  |  |  |  |  |  |  |  |  |  |  |
| JA. De la Torre | Unemployed | 16 - 35 |  |  |  |  |  |  |  |  |  |  |  |  |  |  |  |  |  | 35 |  |

CUD: Cannabis Use Disorder; NEET: Not in Employment, Education or Training

**Appendix G - NEET subgroups description**

Table G.1 – NEET subgroups description in articles with identified-NEET

| **Author** | **Date** | **Subgroup of NEET** | **(N=population)** | **Substance use** | **Type of comparison** | **OR** | **95% CI** | |
| --- | --- | --- | --- | --- | --- | --- | --- | --- |
| CH. Stewart | 2017 | Unemployed | (N= 371) | Current tobacco use | Unemployed NEET vs Non-NEET | 2.34 | [1.85 | 2.96] |
|  |  | Economically inactive* | (N =380) |  |  |  |  |  |
| M. Basta | 2019 | Long terms NEET | (N=82) | Current tobacco use | Long term NEET vs Short Term NEET | 1.06 | [0.64 | 1.76] |
|  |  | Short terms NEET | (N=226) |  |  |  |  |  |
|  |  | Older NEET (23-24) | NA |  |  |  |  |  |
|  |  | Young NEET (below 23) | NA |  |  |  |  |  |
| C. Tanton | 2021 | Unpaid work for own/relative's business | (N=6) | Current tobacco use |  |  |  |  |
|  |  | Waiting to take up paid work already obtained | (N=28) |  |  |  |  |  |
|  |  | Unemployed, looking for work/training | (N=207) |  |  |  |  |  |
|  |  | Temporarily sick/disabled | (N=15) |  |  |  |  |  |
|  |  | Long-term sick/disabled | (N=17) |  |  |  |  |  |
|  |  | Looking after home or family | (N=129) |  |  |  |  |  |
|  |  | Doing something else | (N=27) |  |  |  |  |  |
| L. Rodwell | 2018 | NEET Parenting | (N=24) | Cannabis use | NEET Parenting vs Not NEET parenting | 1.45 | [0.54 | 3.90] |
|  |  | NEET Not Parenting | (N=141) |  |  |  |  |  |

CI: Confident Interval; NEET: Not in Employment, Education or Training; NA: Not Available; OR: Odds Ratio

**Appendix H - Sensitivity and subgroup analyses**

Figure H.1 - Subgroup analysis of current tobacco use prevalence: NEET population compared to non NEET.

Figure H.2 - Subgroup analysis of current tobacco use prevalence: NEET population compared to employed youth.

Figure H.3 - Subgroup analysis of current tobacco use prevalence: NEET population compared to students

Figure H.4 - Subgroup analysis of current tobacco use prevalence: NEET women compared to non-NEET women.

 Figure H.5 - Subgroup analysis of current tobacco use prevalence: NEET men compared to non-NEET men.

Figure H.6 - Subgroup analysis of current tobacco use prevalence: NEET women compared NEET men.

Figure H.7 - Sensitivity analysis of cannabis use prevalence: NEET population compared to non-NEET.

Figure H.8 - Subgroup analysis of cannabis use prevalence: NEET population compared to employed youth.

Figure H.9 - Subgroup analysis of cannabis use prevalence: NEET population compared to students.
